# Photodynamic therapy: research progress and paradigm evolution from mechanism innovation to clinical breakthrough in cancers

**DOI:** 10.4103/mgr.MEDGASRES-D-25-00315

**Published:** 2026-05-19

**Authors:** Yongsheng Cui, Tingting Zhang, Yiwei Du, Kang Guo, Changbin Wang, Yucong Li, Jingzhong Li, Xiaokun Hu

**Affiliations:** 1 Department of Oncology, Shengli Oilfield Central Hospital, Dongying, Shandong Province, China; 2 Department of Interventional Medicine Center, The Affiliated Hospital of Qingdao University, Qingdao, Shandong Province, China

**Keywords:** cancer therapy, clinical translation, combination therapy, immunotherapy, nanomedicine, photodynamic therapy, photosensitizer, reactive oxygen species, tumor hypoxia, tumor microenvironment

## Abstract

**Facts**

1. Photodynamic therapy (PDT) is a minimally invasive anticancer modality based on the light-mediated activation of photosensitizers to generate reactive oxygen species and induce tumor cell death.
2. PDT has demonstrated clinical efficacy in treating various tumor types, including esophageal cancer, gastric cancer, lung cancer, skin cancer, bladder cancer, cervical cancer, ovarian cancer, and glioblastoma.
3. The antitumor effects of PDT are mediated not only by direct tumor cell killing but also by vascular damage, immunogenic cell death, and activation of antitumor immune responses.
4. Nanotechnology-based photosensitizer delivery systems and tumor microenvironment modulation strategies have improved the targeting, oxygenation, and therapeutic efficacy of PDT.
5. Combination strategies that integrate PDT with chemotherapy, radiotherapy, targeted therapy, and immunotherapy are an important way to enhance clinical efficacy and overcome treatment resistance.

**Open Questions**

1. How can photosensitizer selectivity, tumor accumulation, and tissue penetration be further improved to enhance PDT efficacy while minimizing off-target toxicity?
2. What are the optimal light parameters, drug-light intervals, and dosimetry standards for different tumor types and treatment settings?
3. How can tumor hypoxia and oxygen depletion during PDT be effectively overcome in deep-seated or poorly vascularized tumors?
4. What is the optimal timing and sequencing of PDT combined with immunotherapy, chemotherapy, or radiotherapy to maximize the antitumor effect?
5. How can large-scale, multicenter clinical trials be designed to standardize PDT protocols and validate their long-term efficacy, safety, and clinical applications?

Photodynamic therapy is a noninvasive anticancer treatment that uses light-activated photosensitizers to generate reactive oxygen species. However, its clinical efficacy is severely limited by tumor hypoxia and rapid oxygen depletion during the type II photochemical reaction. To address these challenges, we synthesized data from randomized controlled trials and meta-analyses published between 2000 and 2025. Our review shows that photodynamic therapy significantly increases survival rates in patients with malignant pleural effusion and achieves high complete response rates in patients with esophageal and gastric cancers. However, a risk-of-bias assessment reveals limitations in study design regarding blinding and sample size. Crucially, we identify gas-mediated therapies as the next paradigm shift. This review discusses emerging strategies to overcome tumor hypoxia and improve the efficacy of photodynamic therapy. These strategies include oxygen-generating nanoplatforms, tumor microenvironment modulation, and combination approaches with chemotherapy, radiotherapy, and immunotherapy. Additionally, we discuss the critical role of sequential immunotherapy and propose standardized dosimetry protocols that report irradiance and drug-light intervals. These protocols aim to facilitate global regulatory harmonization and clinical reproducibility.

## Introduction

Photodynamic therapy (PDT), as an emerging tumor treatment method, has attracted increasing attention due to its strong selectivity, minimal invasiveness, and repeatability.^1^ PDT relies on the production of reactive oxygen species (ROS) by photosensitizers under light exposure, which then causes damage to tumor cells, providing a safe and effective treatment for cancer patients.^2^ In recent years, researchers have conducted extensive studies on the mechanism, indications, and application of PDT in various types of tumors, forming a relatively rich theoretical foundation and clinical practical experience.

The emergence of PDT not only provides new alternatives to traditional surgeries, radiotherapy, and chemotherapy, but also shows promising prospects in achieving synergistic effects when combined with other treatment methods. PDT can effectively improve the quality of life and overall condition of patients, especially in cases of refractory tumors and advanced tumors.^3^ With the advancement of nanotechnology, a diverse range of novel photosensitizers and treatment approaches have been continuously emerging, thereby substantially expanding the application scope and enhancing the therapeutic efficacy of PDT. Although PDT shows promising application prospects in tumor treatment, there are still some problems and challenges in its clinical application process. For instance, the variations in responses of diverse tumor types to photosensitizers, as well as the optimization of treatment parameters to maximize therapeutic efficacy. Therefore, this article aims to review the latest research progress of PDT in tumor treatment, explore its current clinical application status and future development direction, with the expectation of providing more effective treatment strategies for cancer patients.

## Search Strategy

We searched PubMed, Web of Science, and Embase databases for articles published from January 2000 to December 2025. The search terms included “Photodynamic Therapy” combined with “Cancer,” “Clinical Trial,” “Nanotechnology,” and “Immunotherapy.” We prioritized randomized controlled trials, meta-analyses, and high-impact preclinical studies. Exclusion criteria included duplicates, abstracts without full text, and studies lacking clear dosimetric reporting. A total of 205 references were selected for this review.

## History of Photodynamic Therapy

**Figure 1:**
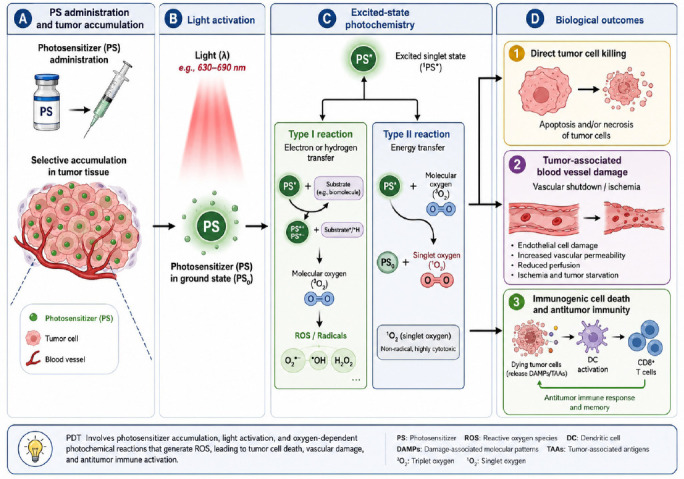
Historical evolution and paradigm shift of PDT. Created with ChatGPT and subsequently reviewed, revised, and finalized using Microsoft PowerPoint 2021 and Adobe Illustrator 2024. 5-ALA: 5-Aminolevulinic acid; H_2_: hydrogen; HpD: hematoporphyrin derivative; NO: nitric oxide; O_2_: oxygen; PDT: photodynamic therapy.

The history of PDT can be traced back to the early 20^th^ century, when scientists first recognized the possibility of generating biological effects when light is combined with photosensitizers. In 1900, German chemist Hermann Staudinger first described the effects of light-sensitive substances on biological systems. With the discovery and development of photosensitizers, PDT gradually entered clinical application. In the 1970s, the broader historical evolution and paradigm shift of PDT are illustrated in **Figure 1**. PDT began to gain prominence in the field of tumor treatment, and its therapeutic effect on skin cancer was particularly well-verified.^4^ During this period, researchers discovered that by irradiating specific wavelengths of photosensitizers with lasers, it is possible to effectively induce apoptosis in tumor cells, thereby achieving non-invasive treatment (**Additional Table 1**).

**Additional Table 1:** Historical evolution of PDT and photosensitizer development

Stage	Key milestone in PDT development	Representative photosensitizers/technologies	Main characteristics and mechanisms
1900–1904	Birth of the PDT concept	Light-sensitive substances; acridine orange	Oscar Raab first observed that light-sensitive substances could induce biological effects under light exposure. Von Tappeiner later introduced the term “photodynamic” and conducted early tumor treatment experiments.
1910–1925	Recognition of hematoporphyrin tumor affinity	Hematoporphyrin	Meyer-Betz reported preferential affinity of hematoporphyrin for tumor tissues, laying the foundation for tumor-selective PDT.
1960s	Emergence of first-generation photosensitizers	HpD; porfimer sodium	HpD was developed by Lipson and colleagues and became the prototype of clinically used first-generation photosensitizers. Porfimer sodium is a purified HpD product composed of porphyrin monomers and polymers. Its mechanism is mainly based on type II photodynamic reactions, generating singlet oxygen to induce tumor cell and vascular damage.
1970s–1980s	Laser technology and early clinical application	Laser systems; optical fiber delivery; HpD-PDT	Advances in laser technology and optical fiber delivery enabled PDT for internal and deep-seated tumors. Early clinical applications included skin, bladder, and other accessible tumors.
1990s	Regulatory approval and development of second-generation photosensitizers	Photofrin; 5-ALA; verteporfin	Photofrin became one of the earliest approved PDT drugs. 5-ALA acts as a precursor of PpIX, which generates singlet oxygen after light activation. Verteporfin, a benzoporphyrin derivative, produces singlet oxygen and selectively damages abnormal neovascular endothelial cells.
1990s–2000s	Expansion of second-generation chlorin-class photosensitizers	Talaporfin sodium; temoporfin	These chlorin-class photosensitizers have improved optical properties and more efficient light activation. Their antitumor effects are mainly mediated by Type II photodynamic reactions, leading to apoptosis, necrosis, and vascular injury.
	Diversified clinical development and indication expansion	Verteporfin; 5-ALA; porfimer sodium; talaporfin	PDT indications expanded from skin and bladder lesions to esophageal cancer, lung cancer, gastric cancer, age-related macular degeneration, actinic keratosis, and other diseases. PDT also began to be explored in combination with radiotherapy, chemotherapy, and immunotherapy.
2000s–2010s	Third-generation photosensitizers and targeted delivery	Targeted photosensitizers; nanoparticle photosensitizers; antibody-, peptide-, aptamer-, and small-molecule-conjugated PSs	Third-generation approaches aim to improve tumor targeting, drug delivery, tissue penetration, and therapeutic selectivity. Although their mechanisms still largely rely on type I and/or II photodynamic reactions, nanocarriers and targeting ligands enable more precise PS delivery and reduce damage to normal tissues.
2010s–present	Precision PDT and multimodal integration	Nanoplatforms; oxygen-generating systems; AIE photosensitizers; image-guided PDT; PDT-immunotherapy combinations	Modern PDT is evolving toward precision and synergistic treatment. Strategies include tumor microenvironment modulation, hypoxia relief, activatable photosensitizers, theranostic nanoplatforms, real-time imaging, dosimetry guidance, and immune activation.

5-ALA: 5-Aminolevulinic acid; AIE: aggregation-induced emission; HpD: hematoporphyrin derivative; PDT: photodynamic therapy; PpIX: protoporphyrin IX; PS: photosensitizer.

Entering the 21^st^ century, PDT has experienced rapid development. The research and development of new photosensitizers have expanded PDT’s application scope in various types of tumors.^5^,^6^ The introduction of this nanotechnology also provides new opportunities for PDT. Researchers have begun to explore the combination of nanoparticles with photosensitizers, aiming to enhance the efficacy of PDT. The increase in clinical trials has provided more data support for the application of PDT, enabling it to demonstrate excellent therapeutic effects in the treatment of various malignant tumors.^7^

Burger et al.^8^ conducted a cost-benefit analysis study in Germany, including 301 patients with non-muscle-invasive bladder cancer, and compared the long-term medical costs of photodynamic diagnostic (PDD)-assisted transurethral bladder tumor resection (TURBT) with those of conventional TURBT. After an average follow-up period of 7.1 years, the annual medical expenses of the PDD group were 168 euros (€2321 *vs*. €2489) lower than those of the conventional group. This was mainly due to a significant reduction in the recurrence rate (32% *vs*. 47%) in the PDD group.

The combined PDT with other treatment methods has also received attention in recent years.^9^ A study has shown that the combined application of PDT with radiotherapy, chemotherapy, targeted therapy, and immunotherapy can significantly enhance the treatment outcome and reduce the risk of tumor recurrence.^10^ With a deeper understanding of the PDT mechanism, researchers have continuously explored more effective treatment methods, bringing new hope to cancer patients.

## Mechanism of Photodynamic Therapy

### Molecular mechanism of photochemical reactions

**Figure 2:**
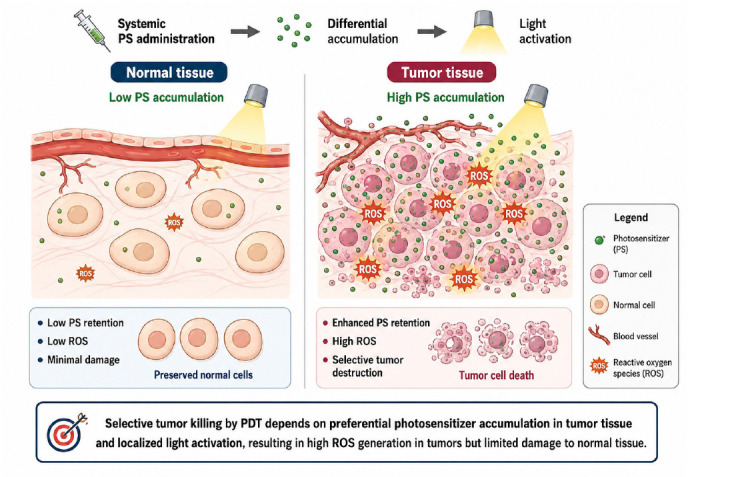
Mechanism of photodynamic therapy. Created with ChatGPT and subsequently reviewed, revised, and finalized using Microsoft PowerPoint 2021 and Adobe Illustrator 2024. DC: Dendritic cell; PS: photosensitisers.

Under the irradiation of specific wavelengths of light, PDT excites the photosensitizers and triggers their photochemical reactions. This process mainly relies on the photosensitizer absorbing light energy, transforming into excited-state molecules, and then combining with reactants such as oxygen to generate ROS, such as singlet oxygen and various free radicals, which are cytotoxic. These ROS not only directly damage tumor cells, but also trigger a series of biochemical reactions, leading to cell apoptosis or necrosis. Type I reaction directly oxidizes biological molecules through free radicals, while type II reaction damages tumor cells and vascular endothelium through singlet oxygen. ROS induces immunogenic cell death (ICD), releasing tumor-associated antigens and damage-associated molecular patterns, activating dendritic cells (DCs) and T cells, initiating adaptive immune responses^11^,^12^ (**Figure 2**).

Different photosensitizers and their concentrations have a significant impact on the efficiency of the photochemical reaction. The selective enrichment of photosensitizers in tumor tissues and the selection of excitation wavelengths directly determine the effectiveness of photochemical reactions. Therefore, studying the photostability of different photosensitizers, the degree of oxygen supply, and the parameters of light exposure, in combination with the microenvironment characteristics of various tumors, will help to further optimize the therapeutic effect of PDT, enhance the killing ability of tumor cells, and improve the safety of the treatment. The development of photosensitizers has evolved through three distinct generations, as summarized in **Additional Table 1**.

### Selective killing of tumors

The selective killing of tumors is an important characteristic of PDT, involving the differential accumulation of photosensitizers between tumor cells and normal cells.^13^ Under the action of the photosensitizer, when specific wavelengths of light are activated, a large amount of harmful ROS is produced. These ROS can effectively kill tumor cells while causing relatively less damage to normal cells. This selectivity makes PDT an ideal treatment method for tumors, especially in cases where traditional radiotherapy and chemotherapy have failed to be effective.

**Figure 3:**
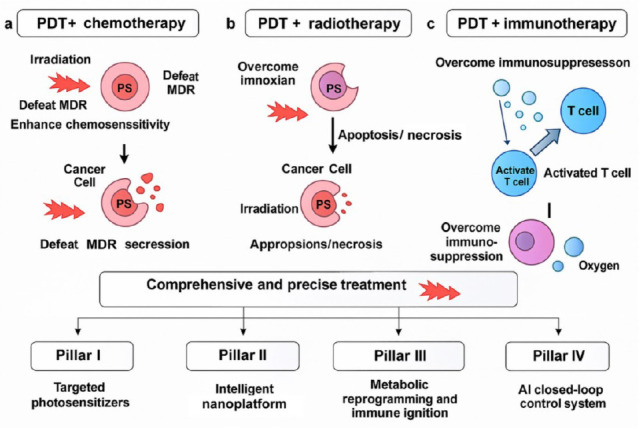
PDT selectively kills tumors. This selective tumor-killing effect of PDT depends on the differential accumulation of photosensitizers (PSs) and localized light activation. After systemic PS administration, only low levels of PS are retained in normal tissue, resulting in limited ROS production and minimal damage to normal cells. In contrast, tumor tissue exhibits enhanced PS accumulation due to abnormal tumor vasculature, increased retention, and factors related to the tumor microenvironment. Upon light activation, the accumulated PS generates high levels of ROS, leading to selective tumor cell destruction while largely preserving surrounding normal tissue. Green dots indicate photosensitizers; red-orange bursts indicate ROS; pink cells indicate tumor cells; pale orange cells indicate normal cells; and red tubular structures indicate blood vessels. Created with ChatGPT and subsequently reviewed, revised, and finalized using Microsoft PowerPoint 2021 and Adobe Illustrator 2024. PDT: Photodynamic therapy; PS: photosensitizers; ROS: reactive oxygen species.

The dual targeting property of PDT stems from the high accumulation of photosensitizers in tumor tissues and the precise positioning of the light source.^14^ This differential accumulation and targeted activation mechanism allows for the selective destruction of malignant cells while sparing normal tissue, as depicted in **Figure 3**. Studies have shown that the particularity of the tumor microenvironment is a key factor for achieving selective killing. Tumor tissues often contain high concentrations of reducing agents and anoxic conditions, which create the conditions for the selective accumulation of photosensitizers.^15^,^16^,^17^ For instance, certain photosensitizers exhibit stronger effects in low-oxygen environments, while they show lower activity in normal tissues. The specific receptor expression on the surface of tumor cells also provides the possibility for the targeted delivery of photosensitizers. By binding to specific target molecules, the photosensitizer can achieve a higher concentration within tumor cells, thereby enhancing its therapeutic effect. A new porphyrin photosensitizer developed by Inai et al.,^18^ when used in a concentration ratio of 5.7:1 to tumor/normal tissue, combined with 635 nm laser irradiation, limits the diffusion range of singlet oxygen to the tumor microenvironment (< 0.2 mm), thereby reducing the damage rate of the surrounding normal tissue to 4.2%. Majernik et al.^19^ confirmed through the chicken embryo chorionic membrane model that hyperoside-mediated PDT selectively inhibits tumor angiogenesis. Zhang et al.^20^ developed a bladder cancer-specific DNA aptamer-modified nanocomposite, which achieved local oxygen concentration enhancement and precise photodynamic killing by targeting the delivery of catalase and the photosensitizer chlorin e6 (Ce6) to the tumor site.

## Clinical Application of Photodynamic Therapy

**Figure 4:**
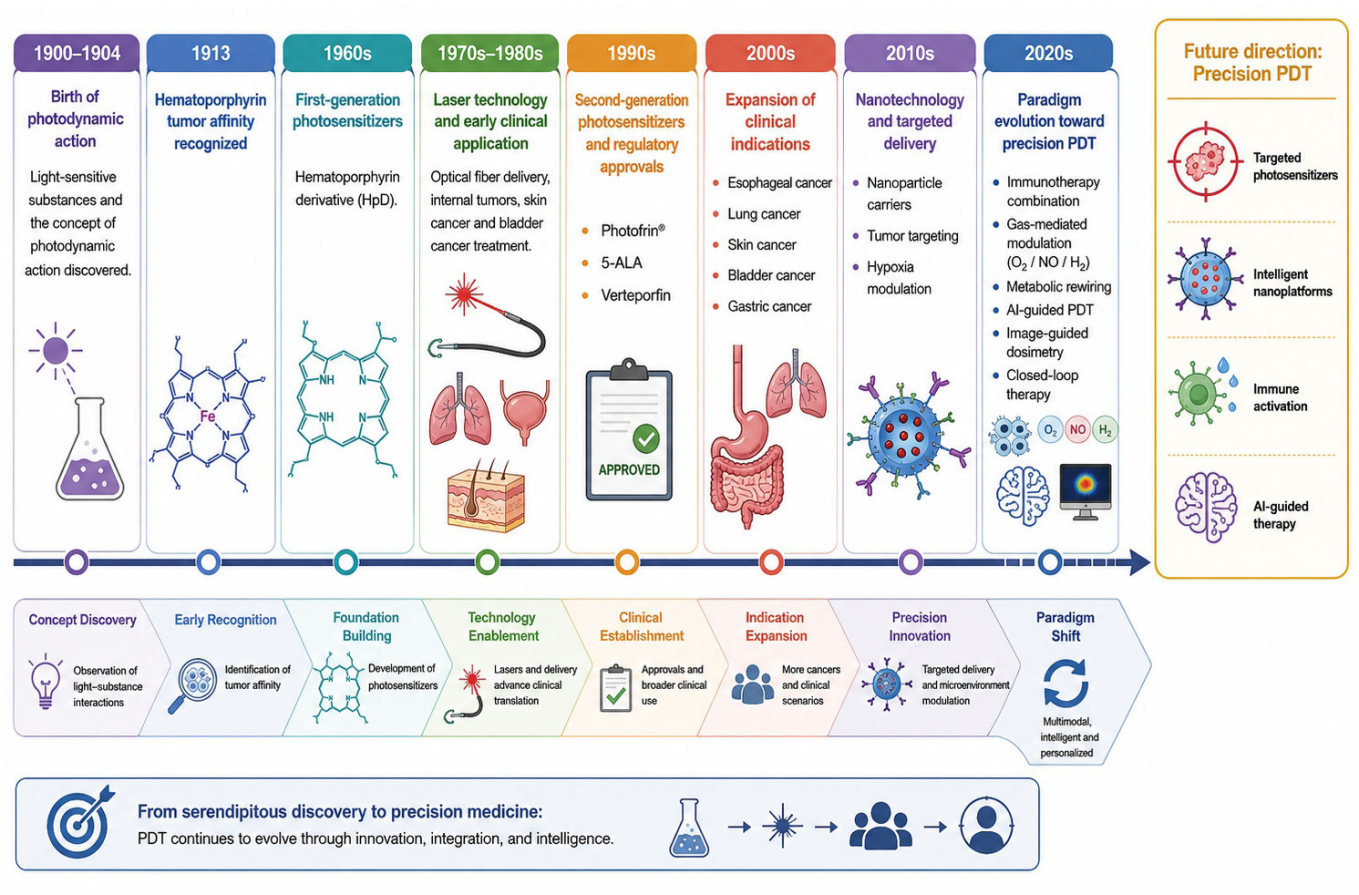
Clinical application landscape of PDT across major tumor types. Created with ChatGPT and subsequently reviewed, revised, and finalized using Microsoft PowerPoint 2021 and Adobe Illustrator 2024. NMIBC: Non-muscle invasive bladder cancer; PDT: photodynamic therapy.

Despite the challenges of hypoxia and light penetration, PDT has established a niche in oncology. To provide a systematic overview of its clinical utility, we have synthesized the key clinical parameters, including photosensitizer dosage, light dosimetry, and efficacy endpoints, across major solid tumors in **Additional Table 1**. **Figure 4** summarizes its major tumor indications, representative clinical roles, and key therapeutic advantages, including minimal invasiveness, selective tumor killing, repeatability, and compatibility with multimodal treatment.

### Digestive system neoplasm

#### Esophagus cancer

In the treatment of esophageal cancer, PDT can effectively alleviate patients’ swallowing difficulties and improve their quality of life, especially in cases where surgery is not possible or where the effects of radiotherapy and chemotherapy are poor, demonstrating a promising clinical application prospect.^21^,^22^

Zeng et al.^23^ demonstrated that the overall effective rate of Hiporfin in treating advanced obstructive esophageal cancer was 78% (25/32). The score for dysphagia decreased from 3.43 ± 0.73 to 1.79 ± 0.53 (*P* < 0.001), and there was no severe skin phototoxicity. Amanuma et al.’s retrospective study^24^ demonstrated that for esophageal squamous cell carcinoma with local recurrence after radiotherapy and chemotherapy, the local complete response rate of PDT was 68% (23/34), and the 2-year overall survival rate reached 79%, which was significantly superior to traditional salvage treatments.

Furthermore, Litle et al.^25^ conducted PDT with porfimer sodium on 215 patients with advanced esophageal cancer (179 cases of adenocarcinoma and 33 cases of squamous cell carcinoma). The results showed that 85% of the patients experienced significant relief of dysphagia symptoms. They could consume liquid food 1 day after the treatment, and resumed soft food within 2–3 days. The median overall survival period reached 16 months. Moreover, patients with a Karnofsky score of ≥ 80 had a significant survival advantage. From this, in the treatment of esophageal cancer, the second-generation photosensitizers have significantly better efficacy and safety compared to the first-generation ones. The third-generation photosensitizers provide a new direction for the treatment of deep-seated tumors through targeted delivery and immune activation mechanisms.

#### Gastric cancer

The application of PDT in the treatment of gastric cancer can be traced back to the early 1990s. Japan was the first to approve its use for early gastric cancer patients who could not undergo conventional endoscopic treatment.^26^ Nakamura et al.^27^ treated eight lesions in seven cases of early gastric cancer with PDT and all achieved complete remission, with only six cases experiencing mild photosensitivity reactions. A Japanese national survey further showed that, among patients with early gastric cancer with lesions ≤ 2 cm and ulcer scars, the complete response rate of PDT reached 73.7%, with no serious complications reported. With the development of endoscopic techniques (such as endoscopic mucosal resection/endoscopic submucosal dissection), the application of PDT in early gastric cancer has somewhat decreased, but it still holds unique value for advanced gastric cancer that is not suitable for surgery. A case report from Japan shows that an 87-year-old patient with cardia cancer had a significant reduction in tumor size after four sessions of PDT, effectively maintaining the ability to eat until 2 years after non-tumor–related death.^28^

For advanced gastric cancer, Chinese researchers compared 144 patients and found that the complete response rate in the PDT combined with chemotherapy group was 20% (8/41), significantly higher than that in the chemotherapy alone group. Furthermore, the median survival time was extended to 11 months (*vs*. 6 months in the control group), suggesting that combined treatment can significantly enhance therapeutic outcomes.^29^,^30^ Although PDT has the problem of a relatively high recurrence rate after treatment, the research data indicate that when used as an auxiliary treatment in combination with chemotherapy or immunotherapy, it shows potential in improving the effectiveness rate (the overall response rate of the combined chemotherapy group reached 58.5%) and enhancing the quality of life, providing an important treatment option for patients who are not suitable for surgery. With the development of nanotechnology, the application of nanoparticles as new photosensitizers has brought new opportunities to PDT.^31^,^32^ It can enhance the targeting and release efficiency of drugs in tumor tissues, thereby improving the therapeutic effect.^33^ At present, the clinical application of PDT in the treatment of gastric cancer is still in the exploration stage, and more clinical trials are needed to verify its safety and effectiveness. Therefore, combining PDT with other treatment methods and seeking the best treatment plan will be the research focus of future gastric cancer treatment.^34^

#### Colorectal cancer

As early as 1986, the Herrera-Ornelas team first applied PDT to 14 patients with recurrent or residual colorectal cancer, confirming that it could effectively eliminate tumor tissues and alleviate symptoms, and with good safety. Another research data show that compared with traditional radiotherapy and chemotherapy, PDT demonstrates multiple advantages in the treatment of advanced colorectal cancer, which not only increases the overall effective rate increase, but also prolongs the survival period of patients and significantly shortens the hospitalization period.^35^ Furthermore, the incidence of complications, such as hematochezia and incomplete intestinal obstruction, in the PDT group was approximately 43% lower than that in the control group, and the incidence of treatment-related adverse reactions was also reduced by 31%.^36^

However, the efficacy of PDT is limited by multiple factors in the tumor microenvironment across various cancer types.^37^ On one hand, interferon γ produced by PDT can promote T cell activation, but it can also significantly upregulate the expression of PD-L1 in tumor cells, thereby weakening the immune killing effect; on the other hand, the hypoxic microenvironment can also trigger high expression of PD-L1.^38^ In response to these challenges, researchers have innovatively constructed multi-dimensional solutions through nanotechnology. Representative studies in colorectal cancer have shown that the nanocorrelation polymer developed by the He team, when combined with oxaliplatin and the photosensitizer pyrolipid, significantly enhances the recruitment ability of T cells to the tumor.^39^ In addition, the di-methyldioxolane liposomes (IR775@Met@Lip) designed by the Wei team overcame the problem of di-methyldioxolane’s difficulty in crossing the membrane by inhibiting mitochondrial complex I, reducing oxygen consumption, and down-regulating PD-L1.^40^ These findings suggest that, in the current era of rapid nanotechnology development, the research and development of new photosensitizers and combination therapeutic strategies may broadly improve the efficacy of PDT in multiple malignancies, including colorectal cancer.^41^,^42^ The results of a systematic review indicate that the complete remission rate for colorectal cancer patients treated with PDT is 40%, and the symptom improvement rate exceeds 50%. The combined treatment approach shows potential in prolonging survival and improving quality of life.^43^,^44^ These advancements indicate that the PDT combined with immune checkpoint inhibitors based on nanotechnology provides a new direction for overcoming the obstacles in the tumor microenvironment. It may become an important auxiliary treatment method for colorectal cancer in the future.

### Respiratory system tumors

PDT is particularly suitable for patients with early-stage or locally advanced lung cancer. In clinical applications, PDT is usually combined with other treatment methods to enhance the overall therapeutic effect.^45^

### Gynecological cancer

#### Cervical cancer

In the treatment of cervical cancer, PDT can effectively inhibit the growth and metastasis of tumor cells.^46^ Carobeli et al.^46^ indicated that by introducing photosensitizers into the bodies of cervical cancer patients and combining them with laser irradiation, the apoptosis rate of tumor cells can be significantly increased. PDT can also activate the immune cells in the body and enhance the immune response of patients, thereby achieving the goal of further eliminating cancer cells.^47^,^48^

Similar to other solid tumors, PDT for colorectal cancer is also constrained by tumor microenvironment-related factors, including hypoxia, heterogeneous photosensitizer distribution, and adaptive immune resistance.^49^ PDT-induced interferon γ can promote T cell activation; however, it may also upregulate PD-L1 expression in tumor cells, thereby attenuating antitumor immune responses. In addition, tumor hypoxia can further promote PD-L1 expression and reduce oxygen-dependent ROS generation, limiting PDT efficacy.^50^ Therefore, although these challenges are not unique to colorectal cancer, they are particularly relevant to colorectal cancer treatment and provide a rationale for combining PDT with nanotechnology-based oxygen modulation and immune checkpoint blockade.

#### Ovarian cancer

The traditional treatment methods of ovarian cancer include surgical removal, chemotherapy and radiotherapy, etc. However, these approaches often come with high recurrence rates and adverse reactions. A clinical study has shown that PDT can effectively reduce the size of tumors and alleviate symptoms in patients with ovarian cancer, thereby improving their quality of life.^51^ Some studies have shown that combining PDT with chemotherapy can enhance the therapeutic effect and reduce the dosage of chemotherapy drugs, thereby minimizing their side effects. Particularly in cases of ovarian cancer patients who are resistant to chemotherapy, PDT demonstrates more significant efficacy.^52^,^53^

Rizvi et al.^54^ verified the synergistic effect of PDT (BPD-MA 1 μM, 690 nm/50 J/cm²) combined with carboplatin chemotherapy in a three-dimensional ovarian cancer model. The average residual tumor fraction in the combined group was 0.26 (0.76 in the single PDT group and 0.95 in the single chemotherapy group), and the expression of the drug-resistant gene ABCG2 was downregulated. Mao et al.^55^ designed a P-glycoprotein inhibitor (Tariquidar) combined with PDT to treat drug-resistant ovarian cancer, which prolonged the survival period of mice and reduced the number of liver metastases.

### Dermatoma

Basal cell carcinoma is the most common type of skin malignancy.^56^ Its occurrence is closely related to ultraviolet exposure. It usually occurs in exposed areas of the skin, such as the face, neck, and arms.^57^ Kessels’s team58 conducted a study on 323 cases of superficial basal cell carcinoma using a double-lighting protocol (applying 5-ALA cream for 3.5 hours and then irradiating twice, with a total energy density of 100 J/cm²). The results showed that the clinical non-recurrence rates at 12, 24, and 48 months were 88.8%, 81.8%, and 77.1%, respectively. The histologically confirmed non-recurrence rates were even higher (90.2%, 85.4%, and 81.8%), indicating that prolonging the light exposure time can significantly improve long-term efficacy.

In clinical applications, PDT has several advantages in the treatment of basal cell carcinoma.^59^ For instance, it causes less damage to the surrounding normal tissues during the treatment process, and the recovery is fast after the surgery, with a low rate of scar formation.^60^ The combined treatment strategies of PDT, such as its combination with immunotherapy, can significantly enhance the anti-tumor effect and promote the immune response of the body.^61^

### Nervous system neoplasms

Glioblastoma (GBM) is the most common and aggressive primary brain tumor.^62^ It typically presents with rapid growth and extensive infiltration, and is highly heterogeneous, making its treatment extremely complex. PDT shows excellent prospects in the combined treatment of GBM.^63^,^64^

Coupiemie’s team^65^ confirmed through in vitro experiments that the PDT treatment with 5-ALA has a good therapeutic effect on GBM. 5-ALA-PDT kills GBM cells through the receptor-interacting protein 3-dependent necrosis pathway. The multicenter clinical study conducted by Mahmoudi et al.^66^ demonstrated that the median overall survival time of GBM patients treated with 5-ALA-PDT combined with surgery was significantly longer than that of the surgical-only group, and the rate of complete resection under fluorescence guidance was also improved. A meta-analysis found that PDT combined with fluorescence navigation could increase the 1-year survival rate of GBM patients to 59%, confirming the clinical efficacy value of PDT.^67^

However, clinical application faces two major hurdles: limited light penetration and the hypoxic tumor microenvironment. To address deep-seated tumors, interstitial PDT has been developed. This technique involves the stereotactic implantation of cylindrical diffusing fibers directly into the tumor mass. A recent study utilizing 5-ALA-guided interstitial PDT has shown that precise fiber placement (10–15 mm spacing) can induce significant necrosis in non-resectable GBMs.^68^

Furthermore, to counteract the “vascular shutdown” effect caused by rapid oxygen depletion, Metronomic PDT strategies are gaining attention. Unlike high-irradiance acute PDT, metronomic PDT employs continuous low-irradiance light (< 10 mW/cm²) delivered over extended periods (hours to days) via implantable devices. This slow infusion of photons matches the rate of oxygen replenishment from capillaries, preventing acute hypoxia and inducing apoptosis rather than necrosis, thereby reducing the risk of severe cerebral edema.^69^,^70^

### Urinary system tumors

Recent research has shown that PDT demonstrates excellent efficacy in the treatment of bladder cancer, especially in the management of postoperative recurrence and refractory cases.^71^ Compared with traditional treatments, PDT has the advantages of less trauma, fewer complications, and higher safety in repeated use, which makes it an ideal supplementary or alternative option in the clinical application of bladder cancer.^72^,^73^

As early as 1987, Prout’s team^74^ began clinical exploration in this field, and for the first time applied hematoporphyrin derivatives to the PDT treatment of superficial bladder transitional cell carcinoma. This pioneering study confirmed the clinical feasibility of this therapy. Since the 21^st^ century, with the development and application of new-generation photosensitizers such as 5-ALA and its derivatives HAL, the selectivity and accuracy of PDT technology in the diagnosis (PDD) and treatment of bladder cancer have achieved a qualitative improvement.^75^,^76^ Li et al.^77^ conducted a systematic meta-analysis by integrating data from 28 studies and 1287 patients (76% of whom were bacillus Calmette-Guérin treatment failures). They found that the 12-month tumor-free survival rate after PDT treatment could reach 68%, and the risk of serious complications was not increased. This indicates that PDT can be an effective option as a second-line treatment.

PDT induces several antitumor effects that are broadly observed across different tumor types, including ROS generation, mitochondrial apoptosis, vascular damage, and immunogenic cell death. In bladder cancer models, these general mechanisms have also been demonstrated. For example, Ce6-mediated PDT increased intracellular ROS levels in T24 and 5637 bladder cancer cells, inhibited superoxide dismutase activity, downregulated Bcl-2 expression, and activated the mitochondrial apoptotic pathway, thereby increasing tumor cell apoptosis.^78^,^79^

The latest clinical data show that 5-ALA-PDT combined with TURBT for treating patients with non-muscle-invasive bladder cancer can significantly reduce the 1-year recurrence rate to 22%. This data demonstrates a significant advantage over the 40–80% recurrence rate of TURBT alone, fully demonstrating the synergistic effect of multimodal treatment.^80^

### Contraindications

Photosensitizers are an essential component in PDT. Allergic reactions of patients to these components may cause serious side effects and even be life-threatening. Some patients may experience a reduction in the efficacy and safety of the photosensitizer due to the use of specific medications or their existing history of adverse reactions, and such factors also need to be carefully considered.

The type and location of the tumor also affect the applicability of PDT. For instance, deep-seated tumors or those located in concealed areas (such as pancreatic cancer, liver cancer) may not be able to achieve sufficient concentrations of photosensitizers due to the limited penetration depth of light.^81^ This can reduce the therapeutic effect. For patients with such tumors, prior to PDT treatment, a careful assessment of the benefits for the patients should be conducted. When there are important structures (such as large blood vessels and nerves) surrounding the tumor, the use of PDT may cause damage to these important structures, leading to serious complications. Therefore, it is necessary to avoid this.

If patients have severe conditions such as severe heart and lung dysfunction, or liver and kidney dysfunction, they may not be able to tolerate the physical burden caused by PDT. Patients with porphyria should avoid using 5-ALA (as it can lead to liver damage due to protoporphyrin IX accumulation), as well as those with photosensitive skin conditions.

## Synergistic Effects of Photodynamic Therapy

**Figure 5:**
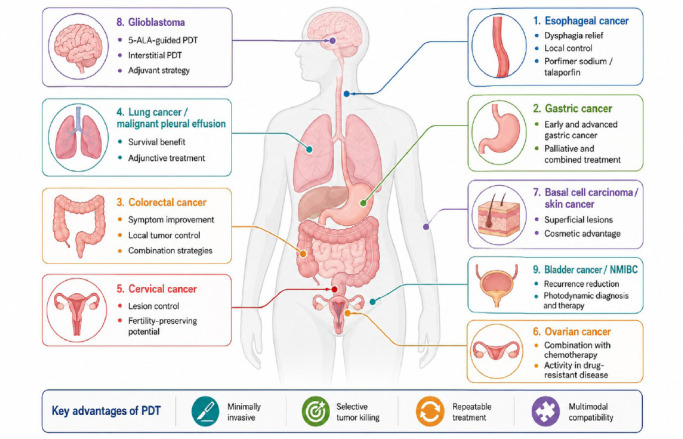
The synergy of PDT with chemotherapy, radiotherapy, and immunotherapy. It illustrated the clinical application landscape of PDT in multiple tumor types, including esophageal cancer, gastric cancer, colorectal cancer, lung cancer/malignant pleural effusion, cervical cancer, ovarian cancer, basal cell carcinoma/skin cancer, glioblastoma, and bladder cancer/non-muscle-invasive bladder cancer (NMIBC). PDT has been explored for local tumor control, symptom relief, recurrence reduction, fertility preservation, cosmetic benefit, and combination therapy with chemotherapy, radiotherapy, immunotherapy, or surgery. The key advantages of PDT include minimal invasiveness, selective tumor killing, repeatable treatment, and multimodal compatibility. Created with ChatGPT and subsequently reviewed, revised, and finalized using Microsoft PowerPoint 2021 and Adobe Illustrator 2024. MDR: Multidrug resistance; PDT: photodynamic therapy; PS: photosensitizers.

As illustrated in **Figure 5**, this multimodal approach spanning chemotherapy, radiotherapy, and immunotherapy overcomes single-agent resistance and enhances comprehensive treatment outcomes.

### Photodynamic therapy combined with chemotherapy

The combined application of PDT and chemotherapy has received extensive attention in recent years. The combined treatment helps overcome the drug resistance of chemotherapy and improves the overall efficacy of tumor treatment. It shows promising prospects in combating various malignant tumors.^82^,^83^

Wang et al.^30^ used hematoporphyrin derivative-PDT combined with chemotherapy to treat patients with advanced gastric cancer. The median survival period of the patients was significantly extended to 11 months (compared to 6 months in the traditional chemotherapy-alone group), and the relief rate of nausea and abdominal pain symptoms reached 80%. In clinical studies, the application of PDT combined with chemotherapy has demonstrated significant synergistic effects in various tumor types. For instance, when treating tumors such as lung cancer and gastric cancer, the combined use of PDT and chemotherapy drugs can effectively enhance the sensitivity of tumor cells to chemotherapy drugs.^84^ PDT may enhance the sensitivity of tumor cells to chemotherapy by inducing ROS-mediated cytotoxic stress, modulating the tumor microenvironment, partially alleviating tumor hypoxia, and improving drug penetration or tumor cell susceptibility to cytotoxic agents.^84^,^85^ In addition, PDT-induced immunogenic cell death can promote the release of tumor-associated antigens and damage-associated molecular patterns, activate DCs and T cell-mediated antitumor immunity, and may provide an additional rationale for combining PDT with chemo-immunotherapeutic strategies.^5^,^86^

### Photodynamic therapy combined with immunotherapy

The combination of PDT and immunotherapy can achieve synergistic antitumor effects through multiple mechanisms.^5^,^85^ PDT generates ROS, induces tumor cell damage, and promotes ICD, thereby increasing the release of tumor-associated antigens and damage-associated molecular patterns. These events can enhance DC activation, antigen presentation, and T cell-mediated antitumor immune responses.^86^,^87^ When combined with immune checkpoint inhibitors, PDT may help overcome the immunosuppressive tumor microenvironment and further improve antitumor efficacy.^88^

A study reported that PDT combined with nivolumab in patients with recurrent esophageal cancer after stent implantation resulted in an overall survival of 11 months, which was longer than that reported with conventional chemotherapy.^89^ In a colorectal cancer CT26 model, the combination of nano-coordination polymers, PDT using pyrolipid as the photosensitizer, and PD-L1 blockade reduced tumor volume and inhibited lung metastasis.^39^

The timing of PDT combined with immunotherapy is also important. PDT-induced ICD and interferon γ release can promote T cell activation, but may also induce adaptive PD-L1 upregulation in tumor cells. Therefore, sequential immune checkpoint blockade after PDT may be more effective than simultaneous treatment. Li et al.^90^ showed that administration of PD-1 inhibitors 24 hours after PDT maximized immune activation compared with concurrent treatment. At this time point, CD8^+^ T cell infiltration in the tumor microenvironment reached 38%, compared with 25% in the concurrent treatment group, suggesting that optimization of the therapeutic window is essential for enhancing the synergy between PDT and immunotherapy.

In addition to immune checkpoint blockade, PDT may also synergize with DC-based vaccines and other immune-modulating strategies. PDT-induced tumor lysates can serve as a source of tumor-associated antigens for loading DC vaccines, thereby enhancing antigen presentation and downstream T cell responses.^91^,^92^ Vedunova et al.^92^ reported that DC vaccines loaded with PDT-induced tumor lysates prolonged survival in a glioma model and increased the proportion of Th17 cells. Moreover, this preclinical study suggests that PDT-DC vaccine strategies combined with anti-cytotoxic T-lymphocyte antigen 4 antibodies may further enhance CD8^+^ T cell infiltration and antitumor immunity.

The combination of PDT and immune checkpoint blockade therapy has also demonstrated strong synergistic potential.^93^,^94^ Zheng et al.^95^ designed a near-infrared II photosensitizer that induced ICD and promoted T cell infiltration. When combined with anti-cytotoxic T-lymphocyte antigen 4 antibodies, this strategy reduced melanoma lung metastases in mice. Another study showed that CD276-targeted immune activation induced by PDT increased CD8^+^ T cell infiltration, and its combination with anti-PD-1 treatment prolonged survival in a mouse model.^96^

Overall, PDT combined with immunotherapy represents an important strategy for converting local tumor destruction into systemic antitumor immunity. Future studies should further clarify the optimal timing, sequencing, immune biomarkers, and patient populations for PDT-based immunotherapy combinations.

### Photodynamic therapy combined with radiotherapy and other non-immunotherapeutic strategies

Beyond chemotherapy and immunotherapy, PDT also combines with radiotherapy, nanomedicine-based delivery systems, and local biomaterial-assisted strategies to enhance tumor control and overcome limitations related to light penetration, photosensitizer distribution, and local drug retention. In radiotherapy, PDT can increase oxidative stress and promote apoptosis or necrosis, while radiotherapy may alter tumor vasculature and improve local treatment sensitivity.^12^,^97^,^98^,^99^,^100^,^101^,^102^ Therefore, PDT combined with radiotherapy may provide enhanced local tumor control in selected tumors. Nanomedicine-based delivery systems also provide opportunities to improve PDT efficacy by enhancing photosensitizer accumulation, local retention, and controlled release in tumor tissues.^103^,^104^,^105^,^106^ Xiao et al.^107^ developed a nanofiber-coated scaffold combined with PDT for advanced esophageal cancer. This strategy increased the local drug concentration in the tumor and improved the complete remission rate from 41% with PDT alone to 67%. These findings suggest that biomaterial-assisted local delivery may help overcome limitations related to insufficient photosensitizer retention and local drug concentration.

**Figure 6:**
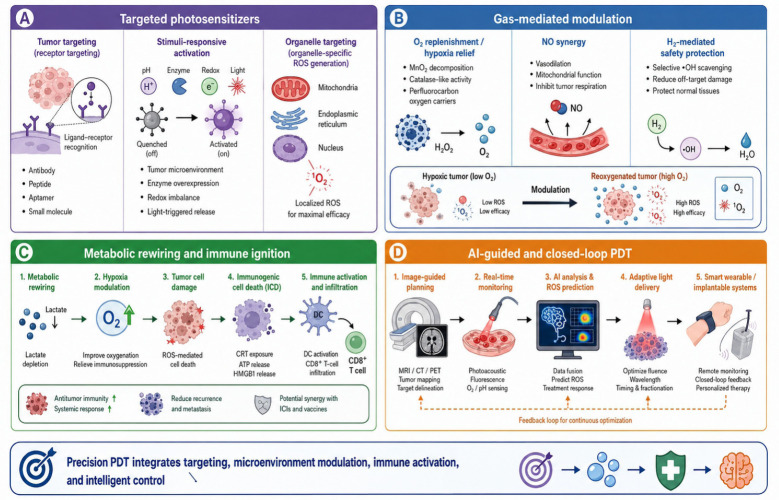
Emerging precision strategies of PDT. (A) Targeted photosensitizers improve tumor selectivity through receptor-mediated targeting, stimuli-responsive activation, and organelle-specific localization, thereby enhancing localized ROS generation. (B) Gas-mediated modulation strategies aim to overcome tumor hypoxia and improve PDT efficacy through oxygen replenishment, NO-mediated vascular modulation, and H_2_-mediated protection against off-target oxidative injury. (C) Metabolic rewiring and immune ignition strategies enhance PDT responses by reducing lactate accumulation, improving tumor oxygenation, inducing ROS-mediated tumor cell damage, promoting immunogenic cell death, and activating dendritic cells and CD8^+^ T cell infiltration. (D) AI-guided and closed-loop PDT integrates image-guided planning, real-time monitoring, ROS prediction, adaptive light delivery, and wearable or implantable systems to optimize treatment precision. Together, these approaches support the transition of PDT toward targeted, microenvironment-responsive, immune-activating, and intelligent cancer therapy. Created with ChatGPT and subsequently reviewed, revised, and finalized using Microsoft PowerPoint 2021 and Adobe Illustrator 2024. DC: Dendritic cell; H_2_: hydrogen; ICD: immunogenic cell death; ICIs: immune checkpoint inhibitors; NO: nitric oxide; PS: photosensitizer; ROS: reactive oxygen species.

Precision PDT is evolving from conventional photosensitizer-light activation toward targeted, microenvironment-responsive, immune-activating, and intelligent therapeutic systems. The major emerging strategies, including targeted photosensitizers, gas-mediated modulation, metabolic rewiring and immune ignition, and artificial intelligence-guided closed-loop PDT, are summarized in **Figure 6**.

**Figure 7:**
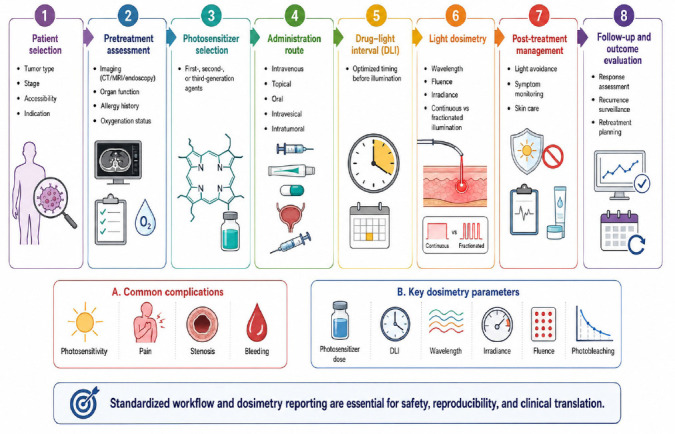
Standardized clinical workflow, dosimetry considerations, and safety management in PDT. Created with ChatGPT and subsequently reviewed, revised, and finalized using Microsoft PowerPoint 2021 and Adobe Illustrator 2024. DLI: drug–light interval; PDT: photodynamic therapy.

## Clinical Approval of Photodynamic Therapy

The U.S. Food & Drug Administration has cleared 6 systemic or topical photosensitizers and > 30 matched light-delivery devices since 1995 (**Additional Table 2**).^24^,^29^,^58^,^67^,^77^ The first-generation hematoporphyrin derivative (Photofrin, Concordia) remains the only topical photosensitizer indicated for both esophageal and endobronchial obstructive tumors as well as high-grade glioma under a humanitarian device exemption. However, its 2–4-week skin phototoxicity window and 68% rate of mild photosensitivity reactions have limited adoption to ~1200 cases per year nationwide. Second-generation agents achieved shorter phototoxicity. Verteporfin (Visudyne, Bausch & Lomb) for age-related macular degeneration was the first office-based intravenous PDT (1999). 5-Aminolevulinic acid 20% topical solution (Levulan Kerastick®, DUSA Dermatology, Billerica, MA, USA) received two supplemental New Drug Applications in 2023, expanding actinic keratosis to full-face/scalp “field therapy,” catalyzing a > 25% year-on-year volume growth in dermatology practices. Rakuten Medical’s RM-1929 (anti-EGFR-IR700 antibody–dye conjugate) completed a phase III open-label randomized trial (NCT03769506) in 2024 Q4; the BLA is under U.S. Food & Drug Administration priority review with a PDUFA goal date of 28 February 2026. If approved, RM-1929 will become the first “targeted PDT biologic” in oncology, potentially resetting the regulatory bar for antibody–photosensitizer conjugates. The Center for Devices and Radiological Health has simultaneously published two final guidelines (March 2025) clarifying 510(k) pathways for artificial intelligence-enabled PDT illuminators and safety reporting for PDT–IO combination protocols, signaling an intention to expedite digital-light-drug combos.

**Additional Table 2:** Key clinical parameters of PDT

Tumor type	PS & protocol	Sample size/design	Light parameter	Efficacy	Adverse event	Reference
Esophageal	Talaporfin (4 h DLI)	*n*=34 (Retrospective)	664 nm; 300 J/cm	L-CR: 69%; 2-yr OS: 79%.	Skin phototoxicity (mild); no stricture	24
Gastric	HpD + Chemo	*n*=144 (Cohort)	630 nm; 150−300 J/cm^2^	ORR: 58.5%; median OS: 11 mon.	Nausea; photosensitivity	29
Basal cell carcinoma (Skin)	Topical 5-ALA-PDT with 2-fold illumination	*n*=323 (Prospective)	630 nm; 37 J/cm^2^	Clearance: 81.8% (48 mon).	Local pain; erythema	58
Glioblastoma	5-ALA + surgery	Meta-analysis	635 nm (interstitial PDT)	1-yr survival: 59%.	Transient edema	67
Bladder	5-ALA + TURBT	*n*=1287 (Meta-analysis)	White/blue light (PDD)	Recurrence: 22% (*vs*. 40−80%).	Frequency; urgency	77

5-ALA: 5-Aminolevulinic acid; Chemo: chemotherapy; DLI: Drug-light interval; HpD: hematoporphyrin derivative; L-CR: local complete response; ORR: objective response rate; OS: overall survival; PDD: photodynamic diagnosis; PDT: photodynamic therapy; TURBT: transurethral resection of bladder tumor.

The European Medicines Agency has granted marketing authorization to three photosensitizers via the centralized procedure. Photofrin (1997),^108^ Visudyne (2000)^109^ and Temoporfin (Foscan®, Biolitec; 2001)^110^ for advanced head-and-neck squamous cell carcinoma under palliative intent. Temoporfin is notable for its 0.1 mg/kg dose and 652 nm activation, allowing 1 cm tissue penetration; nevertheless, national reimbursement remains heterogeneous, Germany’s G-BA rated “minor additional benefit” in 2023, whereas France’s HAS granted ASMR level IV (modest), limiting hospital uptake to about 450 cases a year. Biolotec’s LED-652-CW received Class IIb certification (CE 0476) in 2024 for robotic-assisted interstitial illumination, the first MDR-listed PDT fiber-coupled system enabling MRI-compatible placement.

China’s National Medical Products Administration has approved four domestic Class 1.1 photosensitizers and over 20 matched laser/LED devices. Shanghai Fudan-Zhangjiang dominates the landscape. 5-ALA was first approved in 2007 for condyloma acuminatum, and by 2024, over 3 million cumulative treatments had been carried out, occupying over 90% of the national urogenital wart PDT segment. Hemoporfin, 2017 Class 1.1 certificate for port-wine stain; over 60,000 patients treated, with complete clearance 64% *vs*. 14% pulsed-dye laser in phase III. A phase II solid tumor trial (NCT04129923) was completed in 2024, indicating that Dutaporfin is superior to Photofrin.

Japan’s Pharmaceuticals and Medical Devices Agency granted “Sakigake” designation to RM-1929 in 2023, shortening review to 6 months^111^,^112^; first Asian approval anticipated Q4 2025. NPe6 (Talaporfin sodium, Light-Activated Agent, Meiji) is reimbursed under national insurance for early-stage lung cancer and palliative bile-duct obstruction, with over 8000 cases annually. The amendment in 2024 extended the indication to early esophageal cancer, increasing the eligible population by about 25%.

## Limitations of Photodynamic Therapy

Despite the promising clinical data presented, several intrinsic limitations and methodological biases must temper our conclusions.

### Physiological and physical barriers

As highlighted in colorectal and GBM applications, the efficacy of type II PDT is inherently oxygen-dependent. The hypoxic tumor microenvironment, further exacerbated by oxygen consumption during treatment, remains a critical bottleneck limiting therapeutic success.^15^,^16^ Deep-seated tumors like GBM necessitate invasive interstitial techniques, increasing procedural complexity.^68^

### Biological resistance (adaptive immunity)

A biological limitation identified in recent studies is the reactive upregulation of PD-L1 post-PDT, particularly observed in colorectal and esophageal cancers. This feedback mechanism, triggered by interferon γ, can dampen the immune response, necessitating combination with checkpoint inhibitors to maintain efficacy.^38^,^40^

### Risk of bias and evidence quality

Many cited studies, such as those for cervical or esophageal salvage therapy, are phase I/II trials with small cohorts, limiting statistical power to detect rare adverse events.^24^ Due to the visible nature of light delivery and skin photosensitivity (especially with first-generation photosensitizers), most trials utilize an open-label design.^25^ This introduces potential bias in subjective endpoint reporting (e.g., symptom relief). The lack of standardized protocols across centers hinders the reproducibility of results and meta-analytical synthesis.^113^ Therefore, while PDT demonstrates high response rates, survival benefits should be interpreted with caution until validated by larger, standardized, and ideally blinded phase III randomized controlled trials.

## Summary and Outlook

PDT is evolving from a localized, palliative ablative technique into a precise, immunomodulatory component of multimodal oncology. The era of using simple hematoporphyrin mixtures is passing; we are entering an age of “Intelligent Photochemistry.” The integration of PDT with immunotherapy, specifically the sequential administration of checkpoint inhibitors, has shown the potential to turn “cold” tumors “hot,” offering durable remission beyond the illuminated field (abscopal effect).

With the ongoing harmonization of global regulatory frameworks and the emergence of high-precision third-generation agents, PDT will play an increasingly vital, curative role in the comprehensive treatment of cancer. By converging nanotechnology, gas biology, and artificial intelligence, PDT is poised to break through its historical limitations of hypoxia and depth.

## Additional files:

***Additional Table 1:*** *Historical evolution of PDT and photosensitizer development.*

***Additional Table 2:*** *Key clinical parameters of PDT.*

## Data Availability

*All data generated or analyzed in this study are included in this published article and its additional files.*
